# Clinicopathological and Survival Outcomes of Well-Differentiated Thyroid Carcinoma Undergoing Dedifferentiation: A Retrospective Study from FUSCC

**DOI:** 10.1155/2018/2383715

**Published:** 2018-05-21

**Authors:** Ben Ma, Weibo Xu, Wenjun Wei, Duo Wen, Zhongwu Lu, Shuwen Yang, Tongzhen Chen, Yulong Wang, Yu Wang, Qinghai Ji

**Affiliations:** ^1^Department of Oncology, Shanghai Medical College, Fudan University, Shanghai, China; ^2^Department of Head and Neck Surgery, Fudan University Shanghai Cancer Center, Shanghai, China; ^3^Department of Pathology, Fudan University Shanghai Cancer Center, Shanghai, China

## Abstract

**Background:**

Recently, several studies have reported that dedifferentiation occurs in fatal well-differentiated thyroid cancer (WDTC) cases. This study aimed at investigating the clinicopathological characteristics of WDTC undergoing dedifferentiation.

**Methods:**

A total of 63 WDTC patients harboring dedifferentiated phenotype were enrolled in the study. The Kaplan-Meier method and Cox regression analysis were used to perform survival analyses. Harrell index of concordance (C-index) and Akaike information criterion (AIC) were calculated to compare the predictive value for prognosis among several prognostic classification systems.

**Results:**

The median cause-specific survival (CSS) of patients was 138 months, with the CSS rate of 64.0% and 53.3% at 5 and 10 years, respectively. Presence of the anaplastic thyroid cancer (ATC) phenotype significantly increased the risk of poor CSS (*P* = 0.033), and age was the only independent risk factor for disease progression (*P* = 0.015). The C-index and AIC of the age, grade, extent, size (AGES) prognostic classification system for the CSS were 0.723 and 59.937, respectively.

**Conclusions:**

The presence of dedifferentiated phenotypes can be responsible for the poor outcomes in WDTC patients. The AGES system demonstrates to be an optimal prognostic system for WDTC undergoing dedifferentiation.

## 1. Introduction

In the United States, thyroid cancer has become the fifth most common cancer expected to occur in women in 2017 [1]. Among women before the age of 30 years, thyroid cancer is the most commonly diagnosed cancer in China [2]. Almost the entire change has been attributed to the rapid increase in incidence of well-differentiated thyroid cancer (WDTC), which comprises the vast majority (>90%) of all thyroid cancers [1, 3, 4], including papillary thyroid cancer (PTC) and follicular thyroid cancer (FTC). Though WDTCs usually have good prognoses, some can still develop into aggressive diseases. Recently, several studies have reported presence of poorly differentiated phenotypes in fatal WDTC cases [5–7].

Anaplastic thyroid cancer (ATC) is one of the most aggressive malignancies with a median survival of 5 months and near 100% mortality rate [8] while poorly differentiated thyroid cancer (PDTC) is associated with a 5-year survival rate of 40–70% [5, 9, 10]. ATC and PDTC represent a major clinical challenge because they are refractory to radioiodine therapy and insensitive to traditional chemotherapy and radiotherapy. It has been generally accepted that ATC and PDTC usually develop from the dedifferentiation of differentiated thyroid cancer (DTC), which is supported by that histological concurrence of PDTC/ATC and DTC, and molecular inheritance from DTC to PDTC/ATC has been confirmed in various studies [11–16].

The clinicopathological features of WDTC patients undergoing dedifferentiation are poorly characterized. In the past decade, we have found a cohort of WDTC patients harboring PDTC/ATC at Fudan University Shanghai Cancer Center (FUSCC). Therefore, the present study aimed at investigating the clinicopathological and survival outcomes of WDTC undergoing dedifferentiation.

## 2. Methods

Each patient provided a written informed consent for his/her information to be used for research and stored in the hospital database. All the procedures performed in our study were in accordance with the ethical standards of the FUSCC research committee and the 1964 Helsinki declaration and its later amendments or comparable ethical standards.

### 2.1. Patients

We retrospectively searched for the patients with histopathological diagnosis of WDTC harboring dedifferentiated components, who received surgeries or pathology consultation at FUSCC from January 2004 to December 2016. In the present study, dedifferentiated components were defined as presence of poorly differentiated follicular cell-derived carcinoma or anaplastic carcinoma. The patients included in our study met the following criteria: (1) histopathological confirmation of presence of WDTC, (2) harboring dedifferentiated components at primary site or metastatic sites, and (3) availability of adequate medical records and follow-up outcomes.

### 2.2. Histopathological Review

All hematoxylin and eosin slides of the selected patients were subjected to evaluation by expert pathologists. Tumor size was measured as the maximum dimension of the resected tumor specimen. Extrathyroidal extension (ETE) was divided into two categories: absence of ETE and presence of microscopic or gross ETE. The histological types of tumor were classified according to World Health Organization criteria (2004). PDTC was defined on the basis of the Turin proposal [17]: (1) presence of a solid/trabecular/insular pattern of growth, (2) absence of the conventional nuclear features of papillary carcinoma, and (3) presence of at least one of the following features: convoluted nuclei, mitotic activity ≥ 3 × 10 HPF, and tumor necrosis. In the present study, WDTC undergoing dedifferentiation was defined as primary WDTC with dedifferentiated components at primary or metastatic sites.

### 2.3. Clinicopathological Data

Electronic records were reviewed to collect clinical and histopathological data. The data on clinicopathological features including age, gender, maximum size of tumor, ETE, lymph node metastasis (LNM), distant metastasis (DM), histological subtypes of WDTC and dedifferentiated components, dedifferentiated site, and adjuvant therapy were retrospectively abstracted from patients' records. The selected samples were subjected to repeated evaluation to confirm diagnoses of the above histological features. The patients were evaluated by several prognostic classification systems of thyroid cancer, including European Organization for Research and Treatment of Cancer (EORTC); age, grade, extent, size (AGES); age, metastases, extent, size (AMES); the metastases, age, completeness of resection, invasion, size (MACIS); and tumor, lymph node, metastasis (TNM) stage (the 7th edition) as previously described [18, 19]. The 8th edition of TNM thyroid cancer staging system published by the American Joint Committee on Cancer (AJCC) [20] was also added in our study to be compared with the above staging systems in a prognostic value. The CSS was defined as the time between the date of carcinoma diagnosis by operation and the date of death from cancer. The disease-free survival (DFS) was considered as the duration from the date of carcinoma diagnosis by operation to the date of disease recurrence or progression or death. Patients with no event were censored on the date of the last follow-up.

### 2.4. Statistical Analysis

The continuous data were expressed as the mean ± standard deviation (SD). Categorical results were summarized with frequencies and percentages. *χ*^2^ and Fisher's exact test were used for categorical variables to evaluate their correlations. The Kaplan-Meier method was used to construct CSS and DFS curves, and the univariate survival difference was determined by the log-rank test. Univariate and multivariate analyses were performed to determine risk factors for mortality and disease progression using Cox proportional hazards models calculated by hazard ratio (HR) and 95% confidence interval (CI). Harrell index of concordance (C-index) and Akaike information criterion (AIC) were calculated to compare the predictive value for prognosis among the several prognostic classification systems. A *P* value < 0.05 was considered significant. Statistical analyses were performed using SPSS for Windows (version 22.0; IBM Corp., Armonk, NY) and the R software (version 3.2.5; R Foundation for Statistical Computing, Vienna, Austria). GraphPad Prism (version 6.01; GraphPad Software Inc., La Jolla, Calif) was used to graph survival curves.

## 3. Results

### 3.1. Basic Characteristics of Dedifferentiated Patients

**Figure 1:**
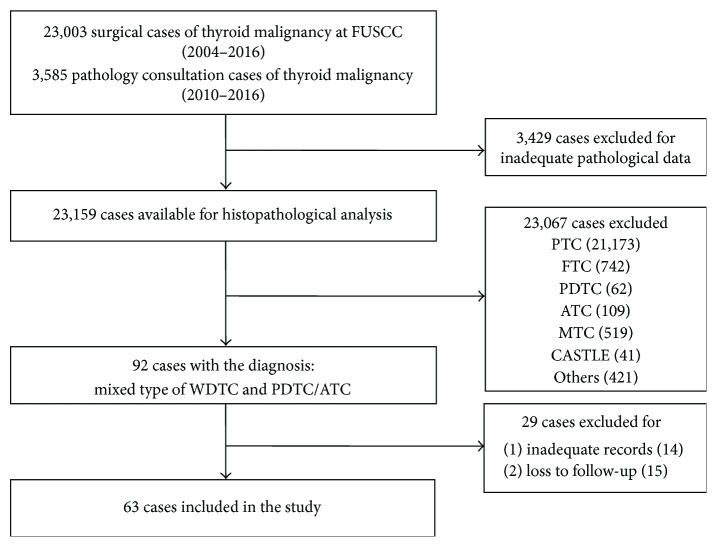
The flow graph of inclusion of WDTC patients harboring dedifferentiated phenotype in our study.

We retrospectively collected and analyzed the records of consecutive WDTC patients with dedifferentiated phenotype confirmed by histopathology who received surgeries or histopathology consultation at FUSCC. The flow graph of inclusion and analysis of study subjects in our study was shown in Figure 1. As shown in Table 1, a total of 63 patients (34 females and 29 males) with an average age of 53.0 ± 15.3 years (range: 15–85 years) were enrolled in our study. The median follow-up time was 52 months (range 8–156 months). Of the 63 WDTC patients, PTC and FTC accounted for 85.7% (54/63) and 14.3% (9/63), respectively. The dedifferentiated phenotype of PDTC was present in 50 patients (79.4%), and the ATC phenotype was observed in 13 patients (20.6%). Dedifferentiation occurred in primary tumor and lymph nodes as well as in distant metastatic sites. Table 1 summarizes the age, gender, tumor size, ETE, WDTC subtypes, dedifferentiated sites and components, TNM stage (the 8th edition), and adjuvant therapy of the patients in detail.

### 3.2. Survival Analyses of WDTC Cases Undergoing Dedifferentiation

**Figure 2:**
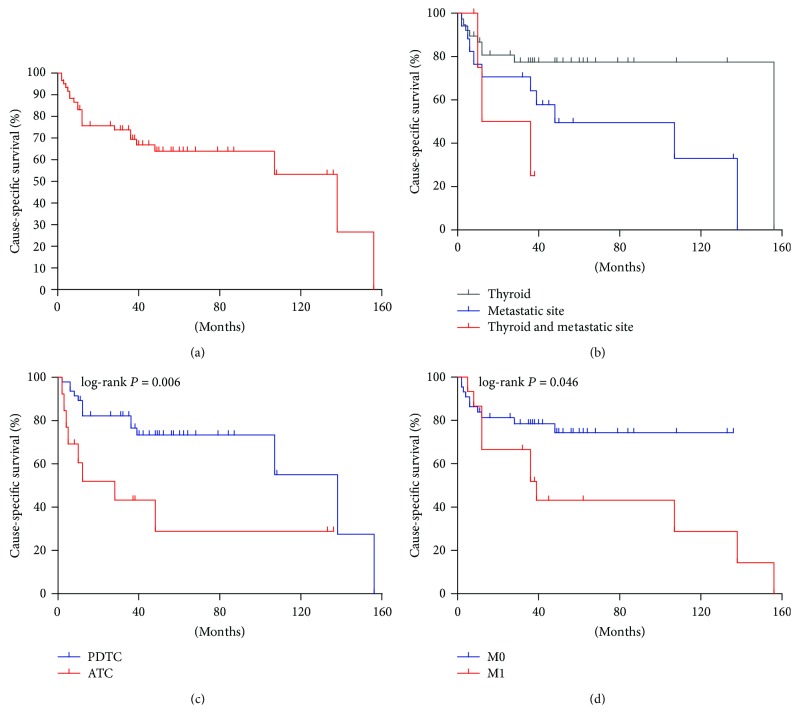
Kaplan-Meier plots of cause-specific survival. (a) The survival plot of all dedifferentiated patients. (b) The survival plot grouped by dedifferentiated sites indicated a significant difference between primary dedifferentiation in thyroid and metastatic region dedifferentiation (*P* = 0.04). (c, d) The survival plots are shown according to dedifferentiated components and distant metastasis (DM), respectively.

The median CSS of patients was 138 months (range 2–156 months), with the CSS rate of 64.0% and 53.3% at 5 and 10 years, respectively (Figure 2(a)). As shown in Table 2, age (HR = 1.041, 95% CI: 1.009–1.076, *P* = 0.013), presence of the ATC phenotype (HR = 3.227, 95% CI: 1.308–7.961, *P* = 0.011, Figure 2(c)), and adjuvant therapy (HR = 3.035, 95% CI: 1.277–7.508, *P* = 0.016) were significantly correlated with decreased CSS in univariate Cox analysis. The further multivariate analysis adjusted by age, dedifferentiated site, dedifferentiated components, DM, and adjuvant therapy indicated that the presence of the ATC phenotype (HR = 3.732, 95% CI: 1.116–12.478, *P* = 0.033) and adjuvant therapy (HR = 2.715, 95% CI: 1.017–7.249, *P* = 0.046) were independent risk factors for the poor CSS.

**Figure 3:**
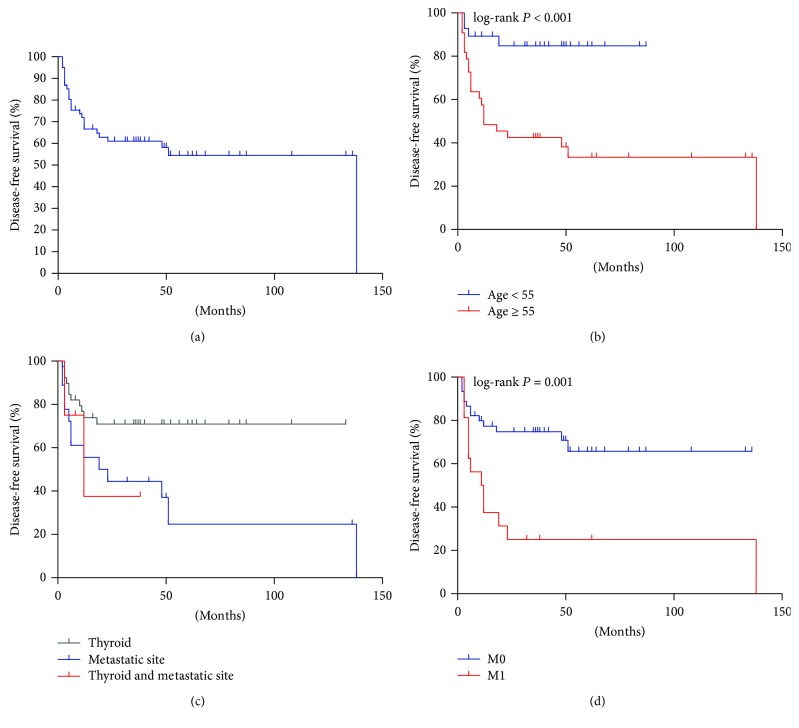
Kaplan-Meier plots of disease-free survival. (a) The survival plot of all dedifferentiated patients. (b–d) The survival plots are shown according to age, dedifferentiated sites, and distant metastasis (DM), respectively. The survival plot grouped by dedifferentiated sites indicated a significant difference between primary dedifferentiation in thyroid and metastatic region dedifferentiation (*P* = 0.011, c).

The DFS curve of patients showed the DFS rate of 53.2% at 5 years in Figure 3(a). The univariate analysis showed that age (HR = 1.045, 95% CI: 1.016–1.076, *P* = 0.002, Figure 3(b)), dedifferentiation of metastatic site (HR = 2.746, 95% CI: 1.210–6.235, *P* = 0.016, Figure 3(c)), and DM (HR = 3.358, 95% CI: 1.517–7.432, *P* = 0.003, Figure 3(d)) significantly increased the risk of decreased DFS in Table 3. After adjustment of age, dedifferentiated site, and DM, age (Table 3, HR = 1.038, 95% CI: 1.007–1.070, *P* = 0.015) was identified as the only independent risk factor for disease progression.

### 3.3. Comparison of Different Prognostic Classification Systems in Dedifferentiated Cases

**Figure 4:**
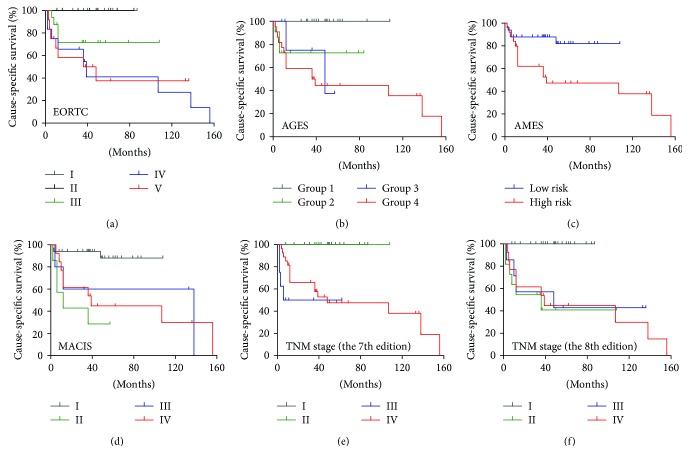
Kaplan-Meier curves of CSS for the prognostic classification systems. (a–f) The Kaplan-Meier curves of CSS according to the prognostic classification systems including EORTC, AGES, AMES, MACIS, the 7th edition TNM, and the 8th edition TNM.

The analyses of evaluation for several prognostic systems including EORTC, AGES, AMES, MACIS, the 7th edition TNM, and the 8th edition TNM were performed to determine whether any of them had a better predictive effect of CSS in dedifferentiated patients.  Table 4 showed the CSS rate of each group of the prognostic systems at 5 years and 10 years and C-index and AIC values for each system. The AGES demonstrated to be a better survival prediction system with 0.723 of C-index and 59.937 of AIC, followed by EORTC (C-index: 0.722, AIC: 69.338), the 8th edition TNM stage (C-index: 0.719, AIC: 60.816), the 7th edition TNM stage (C-index: 0.669, AIC: 68.113), and MACIS (C-index: 0.688, AIC: 68.954), and the AMES had the lowest prediction effect (C-index: 0.650, AIC: 71.915).  Figure 4 showed the Kaplan-Meier curves of CSS according to the above prognostic systems.

### 3.4. Association of Dedifferentiated Features with Clinicopathological Factors

The further analysis of possible factors affecting dedifferentiation components indicated that age ≥ 55 significantly correlated with the presence of the ATC phenotype (Table 5). Additionally, we found that five cases of papillary thyroid microcarcinoma (PTMC) (7.9%) presented dedifferentiation in the thyroid (2/5, 40.0%) and lymph node (3/5, 60.0%), of which 2 cases (40.0%) were observed to be fatal (Table 6).

## 4. Discussion

The present study revealed the dedifferentiation characteristics, the risk factors associated with CSS and DFS, and the optimal prognostic classification system for the 63 WDTC patients harboring dedifferentiated phenotype from FUSCC. The 63 patients were retrospectively selected from a large cohort of thyroid cancer patients at the department of head and neck surgery from FUSCC. To our knowledge, this is the first study that has reported the clinicopathological features and survival outcomes of WDTC harboring dedifferentiated phenotype, which allows us to explain aggressive behaviors and fatal outcomes of some WDTCs in part from the perspective of histological phenotypes.

Consistent with the findings in the previous studies [5, 6, 13], our study confirms that dedifferentiation occurs in both primary tumor and metastatic regions in the histological forms of PDTC and ATC. Considering the significant correlations of age with ATC phenotype and metastatic tumor dedifferentiation, age demonstrates to be a risk factor for aggressive dedifferentiation in this study. And as a result, age is confirmed to be a risk factor for poor DFS in multivariate analysis and decreased CSS in univariate analysis in WDTC patients harboring dedifferentiated phenotype, which is supported by the fact that age is an independent prognostic factor for DTC [20]. The presence of ATC phenotype is an independent risk factor for poor CSS, suggesting that ATC phenotype plays a critical role in determining the patients' prognoses although there is no statistical significance (*P* = 0.137) in the analysis of association between the ATC phenotype and DFS, with the significant effect size (HR = 1.941). In addition, both dedifferentiation of metastatic sites and DM were risk factors for poor CSS and DFS in univariate analysis but could not be statistically significant in multivariate analysis. It is interesting to find that adjuvant therapy after surgery significantly correlates with decreased CSS, which may be explained by insensitivity and increased complications of therapy. Other clinicopathological factors including gender, tumor size, ETE, and histological subtypes of WDTC and LNM are not significantly associated with CSS and DFS in our study.

Due to the widespread use of high-resolution ultrasound and ultrasound-guided fine-needle aspiration, increasing numbers of PTMC patients are diagnosed [21]. Although PTMC usually presents an indolent course, some PTMC patients can still develop to fatal outcomes [7, 22]. In the present study, we found five PTMC cases with dedifferentiation, and 2 patients of them died of cancer disease, indicating that dedifferentiation could be a critical factor for malignant transformation of PTMC into aggressive clinical disease.

The prognostic value of different classification systems can be variable in different types of thyroid cancer [18, 20, 23]. Considering the difference between WDTC harboring dedifferentiated phenotype and DTC, we further analyzed the predictive value of several common classification systems to provide an optimal prognostic system for WDTC patients with dedifferentiated phenotype. As expected, the 8th edition TNM stage shows better predictive value than the 7th edition TNM stage. The AGES system demonstrates to be an optimal system in comparison with the 8th edition TNM stage, EORTC, AMES, and MACIS, which can attribute to the inclusion of histological grade score in the AGES system.

Due to the limited number of WDTC patients harboring dedifferentiated phenotype included in our study, the analyses of clinicopathological outcomes and survival can have statistical bias. Therefore, a large multicenter cohort with long follow-up will be necessary to demonstrate the above outcomes in the future study. Furthermore, the further investigation of molecular biology will be significant to get insights into the mechanism of dedifferentiation of WDTC.

## 5. Conclusions

The presence of dedifferentiated phenotypes can be responsible for the poor outcomes in WDTC patients. The AGES system demonstrates to be an optimal prognostic system for WDTC undergoing dedifferentiation.

## Figures and Tables

**Table 1:** Summary of WDTC patients harboring dedifferentiated phenotype in the FUSCC cohort.

Variables	FUSCC (*N* = 63)	
	*N*	%
Age (years), mean ± SD (range)	53.0 ± 15.3 (15–85)	
Gender		
Male	29	46.1%
Female	34	53.9%
Maximum size (cm), mean ± SD (range)	3.39 ± 2.3 (0.3–8.6)	
ETE		
Yes	15	23.8%
No	48	76.2%
Histological subtypes of WDTC		
PTC	54	85.7%
Classic PTC	46	73.0%
Follicular PTC	2	3.2%
Solid PTC	1	1.6%
PTMC	5	7.9%
FTC	9	14.3%
Dedifferentiated site		
Thyroid	40	63.5%
Lymph node	17	27.0%
Distant site	1	1.6%
Thyroid and lymph node	4	6.3%
Thyroid and distant site	1	1.6%
Dedifferentiated components		
PDTC	50	79.4%
ATC	13	20.6%
LNM		
N0	22	34.9%
N1	41	65.1%
DM		
M0	47	74.6%
M1	16	25.4%
TNM stage (the 8th edition)		
I/II	41	65.1%
III/IV	22	34.9%
Adjuvant therapy		
Yes	15	23.8%
RAI	8	12.7%
Radiotherapy	3	4.8%
Chemotherapy	1	1.6%
Chemoradiotherapy	3	5.3%
No	48	76.2%

WDTC: well-differentiated thyroid cancer; FUSCC: Fudan University Shanghai Cancer Center; SD: standard deviation; ETE: extrathyroidal extension; PTC: papillary thyroid cancer; PTMC: papillary thyroid microcarcinoma; FTC: follicular thyroid cancer; PDTC: poorly differentiated thyroid cancer; ATC: anaplastic thyroid cancer; LNM: lymph node metastasis; DM: distant metastasis; TNM: tumor-node-metastasis; RAI: radioactive iodine.

**Table 2:** Risk factors for decreased cause-specific survival in WDTC patients harboring dedifferentiated phenotype.

Variables	Univariate analysis			Multivariate analysis		
	*P* value	HR	95.0% CI for HR	*P* value	HR	95.0% CI for HR
Gender						
Female		1				
Male	0.134	1.977	0.810–4.822			
Age	**0.013**	1.041	1.009–1.076	0.488	1.014	0.974–1.056
Maximum size	0.113	1.233	0.952–1.597			
ETE						
No		1				
Yes	0.396	0.620	0.205–1.870			
Histological subtypes of WDTC						
FTC		1				
PTC	0.251	2.352	0.545–10.144			
Dedifferentiated site						
Thyroid		1			1	
Metastatic site	0.052	2.531	0.992–6.458	0.337	1.656	0.592–4.633
Thyroid and metastatic site	0.052	3.805	0.991–14.609	0.642	1.510	0.266–8.569
Dedifferentiated components						
PDTC		1			1	
ATC	**0.011**	3.227	1.308–7.961	**0.033**	3.732	1.116–12.478
LNM						
No		1				
Yes	0.322	1.669	0.606–4.4598			
DM						
No		1			1	
Yes	0.056	2.421	0.976–6.005	0.367	1.707	0.534–5.463
Adjuvant therapy						
No		1			1	
Yes	**0.016**	3.035	1.277–7.508	**0.046**	2.715	1.017–7.249

Bold type indicates statistical significance. WDTC: well-differentiated thyroid cancer; HR: hazard ratio; CI: confidence interval; ETE: extrathyroidal extension; FTC: follicular thyroid cancer; PTC: papillary thyroid cancer; PDTC: poorly differentiated thyroid cancer; ATC: anaplastic thyroid cancer; LNM: lymph node metastasis; DM: distant metastasis.

**Table 3:** Risk factors for decreased disease-free survival in WDTC patients harboring dedifferentiated phenotype.

Variables	Univariate analysis			Multivariate analysis		
	*P* value	HR	95.0% CI for HR	*P* value	HR	95.0% CI for HR
Gender						
Female		1				
Male	0.145	1.805	0.816–3.992			
Age	**0.002**	1.045	1.016–1.076	**0.015**	1.038	1.007–1.070
Maximum size	0.351	1.105	0.896–1.364			
ETE						
No		1				
Yes	0.630	1.240	0.517–2.977			
Histological subtypes of WDTC						
FTC		1				
PTC	0.150	2.893	0.682–12.278			
Dedifferentiated site						
Thyroid		1			1	
Metastatic site	**0.016**	2.746	1.210–6.235	0.559	1.312	0.528–3.259
Thyroid and metastatic site	0.250	2.437	0.534–11.109	0.668	1.408	0.296–6.706
Dedifferentiated components						
PDTC		1				
ATC	0.137	1.941	0.809–4.658			
LNM						
No		1				
Yes	0.092	2.323	0.871–6.197			
DM						
No		1				
Yes	**0.003**	3.358	1.517–7.432	0.128	1.914	0.829–4.417
Adjuvant therapy						
No		1				
Yes	0.054	2.249	0.987–5.126			

Bold type indicates statistical significance. WDTC: well-differentiated thyroid cancer; HR: hazard ratio; CI: confidence interval; ETE: extrathyroidal extension; FTC: follicular thyroid cancer; PTC: papillary thyroid cancer; PDTC: poorly differentiated thyroid cancer; ATC: anaplastic thyroid cancer; LNM: lymph node metastasis; DM: distant metastasis.

**Table 4:** Comparison of prognostic classification systems in WDTC patients harboring dedifferentiated phenotype.

Prognostic classification system	Percentage of cause-specific survival		C-index	AIC
	5 years (%)	10 years (%)		
EORTC			0.722	69.338
I-II	94.7	94.7		
III	71.6	71.6		
IV	41.0	27.3		
V	34.6	34.6		
AGES			0.723	59.937
Group 1	100.0	100.0		
Group 2	65.6	65.6		
Group 3	37.5	37.5		
Group 4	42.5	34.0		
AMES			0.650	71.915
Low risk	82.0	82.0		
High risk	43.5	34.8		
MACIS			0.688	68.954
I	88.1	88.1		
II	22.2	22.2		
III	60.0	60.0		
IV	44.9	29.9		
TNM stage (7th)			0.669	68.113
I-II	95.8	95.8		
III	50.0	50.0		
IV	45.9	36.7		
TNM stage (8th)			0.719	60.816
I	95.2	95.2		
II	37.5	37.5		
III	42.9	42.9		
IV	44.9	29.9		

WDTC: well-differentiated thyroid cancer; C-index: index of concordance; AIC: Akaike information criterion; EORTC: European Organization for Research and Treatment of Cancer; AGES: age, grade, extent, size; AMES: age, metastases, extent, size; MACIS: metastases, age, completeness of resection, invasion, size; TNM: tumor, lymph node, metastasis.

**Table 5:** Clinicopathological factors associated with dedifferentiated components.

Parameters	Dedifferentiated components		*P* value
	PDTC	ATC	
Gender			**0.013**
Male	19 (65.5%)	10 (34.5%)	
Female	31 (91.2%)	3 (8.8%)	
Age (years)			**0.009**
<55	28 (93.3%)	2 (6.7%)	
≥55	22 (66.7%)	11 (33.3%)	
Tumor size (cm)			0.554
≤1	6 (85.7%)	1 (14.3%)	
>1	44 (78.6%)	12 (21.4%)	
ETE			0.371
No	39 (81.3%)	9 (18.7%)	
Yes	11 (73.3%)	4 (26.7%)	
DTC subtypes			0.472
PTC	41 (80.4%)	10 (19.6%)	
FTC	9 (75.0%)	3 (25.0%)	
Dedifferentiated site			0.536
Thyroid	33 (82.5%)	7 (17.5%)	
Metastatic site	14 (77.8%)	4 (22.2%)	
Thyroid and metastatic site	3 (60.0%)	2 (40.0%)	
LNM			0.502
N0	17 (77.3%)	5 (22.7%)	
N1	33 (80.5%)	8 (19.5%)	
DM			0.570
M0	37 (78.7%)	10 (21.3%)	
M1	13 (81.3%)	3 (18.7%)	
TNM stage (the 8th edition)			0.142
I-II	37 (84.1%)	7 (15.9%)	
III-IV	13 (68.4%)	6 (31.6%)	

Bold type indicates statistical significance. PDTC: poorly differentiated thyroid cancer; ATC: anaplastic thyroid cancer; ETE: extrathyroidal extension; PTC: papillary thyroid cancer; FTC: follicular thyroid cancer; LNM: lymph node metastasis; DM: distant metastasis; TNM: tumor-node-metastasis.

**Table 6:** Clinicopathological characteristics of PTMC patients with dedifferentiated phenotypes.

Number	Age	Gender	Dedifferentiated site	Dedifferentiated components	ETE	LNM	DM	TNM stage	Cause-specific survival status
1	73	Female	Lymph node	PDTC	No	Yes	No	II	NA
2	31	Female	Thyroid	PDTC	No	No	No	I	Alive
3	47	Female	Lymph node	PDTC	No	Yes	Lung	II	Dead
4	61	Female	Lymph node	ATC	No	Yes	No	II	Dead
5	41	Male	Thyroid	PDTC	No	No	No	I	Alive

PTMC: papillary thyroid microcarcinoma; ETE: extrathyroidal extension; LNM: lymph node metastasis; DM: distant metastasis; TNM: tumor-node-metastasis; PDTC: poorly differentiated thyroid cancer; ATC: anaplastic thyroid cancer; NA: not available.
